# The Circadian Clock and Viral Infections

**DOI:** 10.1177/0748730420967768

**Published:** 2020-11-09

**Authors:** Helene Borrmann, Jane A McKeating, Xiaodong Zhuang

**Affiliations:** Nuffield Department of Clinical Medicine, University of Oxford, Oxford, UK

**Keywords:** circadian rhythm, viruses, COVID-19, virus-clock, virology

## Abstract

The circadian clock controls several aspects of mammalian physiology and orchestrates the daily oscillations of biological processes and behavior. Our circadian rhythms are driven by an endogenous central clock in the brain that synchronizes with clocks in peripheral tissues, thereby regulating our immune system and the severity of infections. These rhythms affect the pharmacokinetics and efficacy of therapeutic agents and vaccines. The core circadian regulatory circuits and clock-regulated host pathways provide fertile ground to identify novel antiviral therapies. An increased understanding of the role circadian systems play in regulating virus infection and the host response to the virus will inform our clinical management of these diseases. This review provides an overview of the experimental and clinical evidence reporting on the interplay between the circadian clock and viral infections, highlighting the importance of virus-clock research.

## Introduction: Circadian Rhythms from the Macroscale to the Microscale

**Figure 1.**
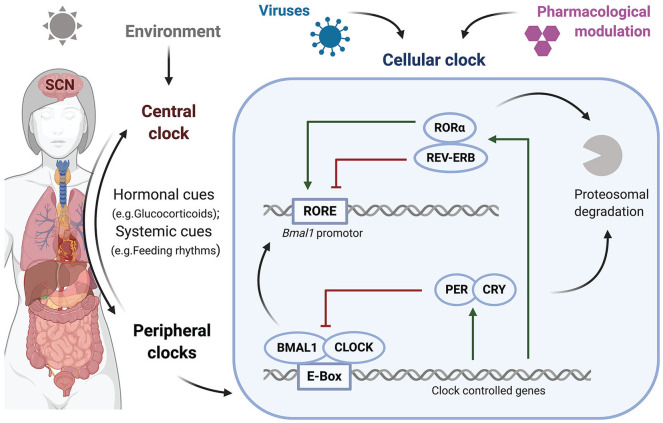
Circadian regulation of the human body. External signals entrain the body’s central clock, the SCN in the brain, which in turn synchronizes peripheral clocks in organs and results in the molecular regulation of each cellular clock. Transcriptional/translational feedback loops coordinate rhythmic gene expression, and proteasomal degradation of the components restarts the system. BMAL1:CLOCK binds to E-Boxes and activates the expression of REV-ERB, RORα, and PER:CRY, which then directly inhibit BMAL1:CLOCK or act on *Bmal1* promoter activity. SCN = suprachiasmatic nucleus; BMAL = brain and muscle ARNT-like 1; CLOCK = circadian locomotor output cycles kaput; ROR = related orphan receptor; PER = Period; CRY = Cryptochrome. Created with BioRender.com

The Earth’s rotation leads to day/night cycles and drives our daily circadian rhythms right down to microscale processes at the cellular level. In mammals, the primary external time cue (zeitgeber) is the light/dark cycle that transduces signals through the retinohypothalamic tract to the suprachiasmatic nucleus (SCN) or “master clock” in the brain. The SCN communicates with the rest of the body to coordinate the circadian rhythm in every tissue (Astiz et al., 2019), allowing organisms to anticipate and adapt to their changing environment (Figure 1). The SCN comprises a complex neuronal network with multiple neural connections across the brain and transmits signals to the autonomic nervous and endocrine systems (Astiz et al., 2019). The influence of the SCN on the autonomic nervous system alters the sensitivity of peripheral tissues to hormones (Buijs et al., 2006). Consequently, the same hormone stimulus may induce different responses dependent on the time of the day (Becker et al., 2019; Garcia-Garcia and Mendez-Ferrer, 2020). The importance of the SCN as the main pacemaker becomes evident as transplantation of a functioning SCN reestablishes the circadian rhythm in genetically arrhythmic mice (Ralph et al., 1990; Sujino et al., 2003); however, other extracellular cues can independently synchronize peripheral tissues.

Within individual cells, the circadian clock machinery is the self-sustaining product of transcriptional/translational feedback loops (TTFLs) consisting of activators and repressors (Figure 1). The two main activators, the basic helix-loop-helix transcription factors CLOCK (circadian locomotor output cycles kaput) and BMAL1 (brain and muscle ARNT-like 1), form heterodimers that bind genomic regulatory elements called E-boxes to regulate circadian gene expression. This complex induces the expression of the repressors PER (Period) and CRY (Cryptochrome), which dimerize and form a negative feedback loop that represses CLOCK:BMAL1-mediated transcription. A second interlocked TTFL regulates BMAL1 expression, comprising the nuclear receptors REV-ERBα and RORα, which bind retinoic acid–related orphan receptor (ROR) response elements (RORE) in the *Bmal1* promoter to activate or inhibit its transcription, respectively (Guillaumond et al., 2005). REV-ERBα and RORα contain E-boxes in their promoters and hence are regulated by BMAL1:CLOCK. Ubiquitination-dependent degradation of these transcription factors resets the TTFL, establishing a 24-h oscillation of the RNA and protein products of these core clock regulators (Takahashi, 2017).

**Figure 2.**
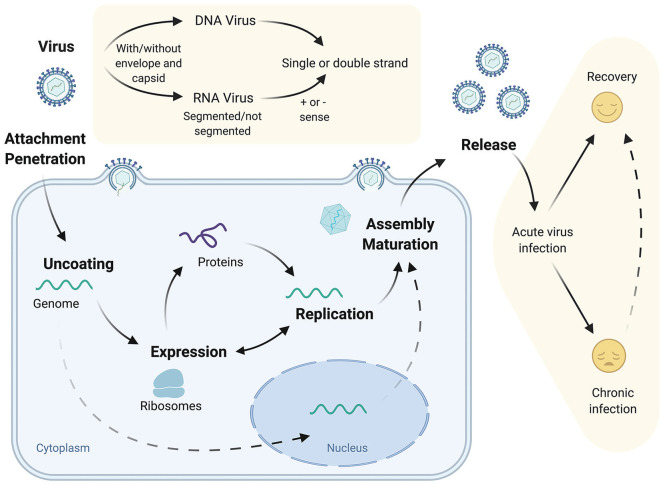
Virus structure, life cycle, and disease outcome. Viruses bear their genetic information as encapsidated RNA or DNA that may be enclosed in a lipid envelope. After the virus entry into the host cell, the genetic material is released, translated, and replicated. For some viruses, their genetic material is imported and replicated in the nucleus. After translation of the viral proteins and replication of the genome, new viral particles are assembled and released to complete the life cycle. Virus infection can be resolved by host immune responses or persist and cause chronic disease. Created with BioRender.com

As obligate parasites, viruses are completely reliant on their hosts for replication and dissemination. The first step of the viral life cycle is its entry into the cell, via binding of the virion to host factors or receptors expressed at the cell surface (Helenius, 2018). After viral particle entry and capsid disassembly, the RNA or DNA genomes are released into the cell and exploit host transcriptional and translational pathways to initiate their replication (Figure 2). In simplistic terms, virus infections may be acute, a “hit-and-run” strategy of virus replication as exemplified by influenza virus, or chronic, establishing long-lasting infection such as hepatitis B virus (HBV), reflecting the balance between the host immune response and viral evasion strategies (Virgin et al., 2009). A recent study in primates uncovered that more than 80% of protein coding genes in various tissues show daily rhythmic expression (Mure et al., 2018), and given the dependency of virus replication on cellular pathways, it is unsurprising that host clock components have been reported to directly or indirectly influence virus replication.

More recently, a disrupted circadian clock has been linked to increasing susceptibility to several pathogen-associated diseases (Bellet et al., 2013; Kiessling et al., 2017; Hopwood et al., 2018; T. Li et al., 2019; Diallo et al., 2020; Kitchen et al., 2020), and the interplay between viral infections and the circadian clock is of increasing interest with an expansion of work in this area (Zhuang et al., 2017; Mazzoccoli et al., 2020). This review provides an overview of host-virus interactions from a circadian perspective and a summary of reported viral interactions with the circadian components (Table 1).

**Table 1.** Overview of virus interactions with the cellular circadian clock.

Virus	Clock Component	Effects and Proposed Mechanisms	References
Dengue virus	BMAL1, REV-ERB	• Modulation of host lipid pathway essential for replication • REV-ERB inhibits stearoyl-CoA-desaturase expression (rate-limiting for infection) • Dengue infection increases the locomotor activity of vector *Aedes aegypti* females	Lima-Camara et al. (2011); Zhuang et al. (2019)
Hepatitis B virus	CLOCK, BMAL1, PER1-3, CRY1,2	• Overexpression of HBx protein disrupts expression of circadian genes • Lower mRNA levels of *Per1-3* and *Cry2* in HCC tissue	Yang et al. (2014)
Hepatitis C virus	BMAL1, REV-ERBPER2,CRY2	• HCV infection upregulates miR-10 which downregulates *Bmal1* via suppressing RORα • Overexpression of *Per2* counteracts HCV replication • Downregulation of *Per2* and *Cry2* in HCV patients • Modulation of host lipid pathway essential for replication • REV-ERB inhibits stearoyl-CoA-desaturase expression (rate-limiting for infection)	Benegiamo et al. (2013); Zhuang et al. (2018, 2019); Horii et al. (2019)
Herpesvirus	CLOCK, BMAL1	• CLOCK activates expression of entry receptor via binding to its promoter • Virus induces BMAL1 expression, and low levels of BMAL1 increase infection • HSV viral protein IPC0 interacts with CLOCK:BMAL1, remodeling of viral chromatin	Kalamvoki and Roizman (2010, 2011); Edgar et al. (2016); Matsuzawa et al. (2018)
Human immunodeficiency virus	CLOCK, BMAL1, REV-ERB, PER2	• CLOCK/BMAL1 drive HIV transcription via E-box in LTR, and REV-ERB decreases HIV promoter activity • Tat decreases amplitude of circadian rhythm • SNPs in *CLOCK* and *Per2* genes alter sleep behavior	Clark et al. (2005); Duncan et al. (2008); Wang et al. (2014); Lee et al. (2015b); Chang et al. (2018); Borrmann et al. (2020)
Influenza virus	BMAL1, CLOCK, REV-ERB,PER2	• Virus-induced *Bmal1* expression • IAV advances *Bmal1, Clock*, and *Rev-erbβ* expression and increases *Per2* amplitude • Low levels of BMAL1 increase viral load • Regulation of infection through time-dependant host tolerance/immune activation	Sundar et al. (2015); Edgar et al. (2016); Ehlers et al. (2018); Sengupta et al. (2019)
Parainfluenza virus type 3	BMAL1	• BMAL1 decreases viral replication	Majumdar et al. (2017)
Respiratory syncytial virus	BMAL1	• BMAL1 decreases viral replication	Majumdar et al. (2017)
Simian immunodeficiency virus	Unknown	• Infection impairs amplitude of circadian rhythm of body temperature and locomotor activity	Huitron-Resendiz et al. (2007)
Zika virus	BMAL1, REV-ERB	• Modulation of host lipid pathway essential for replication • REV-ERB inhibits stearoyl-CoA-desaturase expression (rate-limiting for infection)	Zhuang et al. (2019)

Abbreviations: BMAL = brain and muscle ARNT-like 1; CLOCK = circadian locomotor output cycles kaput; PER = Period; CRY = Cryptochrome; ROR = related orphan receptor; HSV = herpes simplex virus; LTR = long terminal repeat; HCC = hepatocellular carcinoma; SNP = single-nucleotide polymorphism; IAV = influenza A virus.

## Circadian Pathways Shape Viral Infection

### A Role for the Endocrine System in Regulating Viruses

Many factors can synchronize peripheral clocks, including the autonomic nervous system (Becker et al., 2019; Garcia-Garcia and Mendez-Ferrer, 2020), body temperature (Buhr et al., 2010), fasting/feeding cycles (Wehrens et al., 2017; Lewis et al., 2020), and cytokines and hormones (Astiz et al., 2019). The autonomic nervous system is influenced by the circadian clock, and some viruses can infect the peripheral and central nervous system (Koyuncu et al., 2013); however, at the present time, there is no evidence for a direct interaction of viruses, clock, and the autonomic nervous system. The endocrine system is the major route for synchronizing the SCN with peripheral clocks, and glucocorticoid expression is one of the best-studied examples (Oster et al., 2006, 2017; Pezuk et al., 2012). Glucocorticoid receptor signaling shows a bidirectional interaction with the circadian clock (Lamia et al., 2011; Caratti et al., 2018). Therapeutic administration of glucocorticoids (e.g., commonly prescribed cortisol) has been associated with the increased reactivation of HBV (Hatano et al., 2019) and poor clinical outcomes in influenza infection (Tsai et al., 2020). The glucocorticoid dexamethasone was recently shown to reduce the severity of coronavirus disease (COVID-19) (Delaney et al., 2016; Horby et al., 2020). The observation that corticosteroids suppress coronavirus HCoV-229E replication and cytokine production in primary cultures of human nasal and tracheal epithelial cells (Matsuyama et al., 2020; Yamaya et al., 2020) provides a potential mechanism for these clinical observations. Melatonin released from the pineal gland modulates sleep behavior and has been reported to have wide-ranging antiviral activities in virus-induced diseases (reviewed in Anderson and Reiter, 2020). In mouse model systems, melatonin can inhibit the NLRP3 inflammasome (Y. Zhang et al., 2016; Cao et al., 2017; Ma et al., 2018), leading us and others (Shneider et al., 2020) to speculate a role for this inflammatory pathway in COVID-19.

### Circadian Regulation of Host Immunity: Impact on Virus Infection

To anticipate environmental changes and minimize the risk of infection, many immune parameters oscillate throughout the day (reviewed in Man et al., 2016; Scheiermann et al., 2018). Certain functions of the innate immune system depend on the cellular clock, where CLOCK, BMAL1, and REV-ERB regulate essential processes such as the expression of pattern recognition receptors (PRRs) that are involved in nucleic acid sensing during viral infections (Silver et al., 2012, 2018). Mice with latent murine γ-herpesvirus infection received an acute inflammatory challenge while being exposed to repeated chronic diurnal disruption that mimics shift work. The authors noted a reactivation of latent virus, leading to increased viral loads and changes in cytokine and chemokine concentrations in the lung (Trammell and Toth, 2016). Sengupta et al. (2019) showed the importance of natural killer T cells, natural killer cells, and inflammatory monocytes (Ly6c^hi^ monocytes) in the time-of-day dependency of influenza infection. Nguyen et al (2013) showed that BMAL1 regulated the diurnal oscillation of Ly6C^hi^ monocytes. Other respiratory viruses are circadian-dependent: for example, Majumdar et al. (2017) reported that BMAL1 deficiency increased the susceptibility to respiratory syncytial virus (RSV) and PIV3 (human parainfluenza virus type 3) infection. REV-ERB can repress inflammatory responses and chemokine secretion in the context of pulmonary inflammation (Caratti et al., 2018). Genetic disruption of REV-ERB increased endotoxin responses in macrophages and enhanced secretion of the pro-inflammatory cytokine interleukin 6 (IL-6) (Gibbs et al., 2012). When studying macrophage biology, it is relevant to study their different biological functions separately as phagocytosis is not time-of-day dependent, whereas cytokine expression is rhythmic (Geiger et al., 2019). These patterns are also seen for the adaptive immune response, for example, in lymphocyte development and trafficking (reviewed in Scheiermann et al., 2018). Lymphocyte numbers oscillate over the course of the day, with glucocorticoids inducing diurnal T-cell accumulation in lymphoid organs (Shimba et al., 2018). Loss of lymphocyte circadian clocks ablates rhythmic adaptive immune responses important for combating influenza A virus (Druzd et al., 2017). BMAL1 is linked to mitochondrial function and metabolic pathways, which influences the phenotype and activity of immune cells. Rhythmicity of immunometabolism has become a key aspect of immune defense and disease outcomes (Early and Curtis, 2016; Carroll et al., 2019). These studies illustrate how the circadian clock regulates the immune response that impacts viral replication.

### Circadian Regulation of Host Pathways Essential for Virus Infection

The liver is one of the most circadian-regulated organs of the body, with 20% of the transcriptome showing rhythmic expression (R. Zhang et al., 2014), and viruses infecting the liver are likely to be affected by the clock. A recent clinical study showed more rapid hepatitis C virus (HCV) reinfection kinetics following liver transplantation when the surgery was conducted in the morning compared with in the afternoon (Zhuang et al., 2018). In vitro studies showed that HCV infection of circadian-synchronized hepatocytes was linked to rhythmic expression of viral receptors (Zhuang et al., 2019), consistent with the increased infection kinetics observed after liver transplant. In addition, genetic knockout (KO) of *Bmal1* or pharmacological activation of REV-ERB inhibited the replication of HCV and the related flaviviruses, dengue and Zika, by repressing lipid pathways that are essential for their replication (Zhuang et al., 2019).

Identifying host factors that associate with viral proteins can uncover new drug targets. A recent mapping of the interactome of SARS-CoV-2 proteins identified 66 druggable host factors (Gordon et al., 2020). It is worth noting that 30% of these host genes show circadian oscillation (Ray and Reddy, 2020), lending further support for circadian regulation of SARS-CoV-2 replication and potential chronotherapies in treating COVID-19 (Meira et al., 2020; Ray and Reddy, 2020).

Chang et al. (2018) reported an association between peripheral viral RNA levels in human immunodeficiency virus type 1 (HIV-1)-infected patients and the time of sampling, with increased levels of unspliced HIV RNAs associated with BMAL1 expression. Furthermore, CLOCK and BMAL1 overexpression induced HIV transcription via an E-box motif in the HIV long terminal repeat (LTR) (Chang et al., 2018). A recent study found that pharmacological activation of REV-ERB decreased BMAL1 and inhibited HIV LTR activity and viral replication in cell lines and primary CD4 T cells and induced pluripotent stem cell–derived macrophages (Borrmann et al., 2020). Importantly, mutations within the E-box reduced basal LTR activity, highlighting the multifunctional nature of this motif and suggest that circadian factors cooperate with other host transcription factors in regulating HIV replication. Interestingly, additional conserved circadian regulatory elements including the RORE and the glucocorticoid response element were found in the HIV-LTR, begging the question whether HIV replication may be synchronized at certain times of day.

Herpes simplex virus 2 (HSV-2) infection of mice was less severe when infection occurred during the rest phase compared with during the active phase (Matsuzawa et al., 2018). The HSV-2 entry receptor Nectin1 (*Pvrl1*) in mouse and human keratinocytes shows rhythmic expression and is directly regulated by CLOCK. CLOCK silencing decreased *Pvrl1* expression, suggesting a role for CLOCK in regulating HSV-2 infection. Disrupting *Bmal1* enhanced the replication of murid herpesvirus 4 (MuHV-4) and HSV-1 in vivo and in vitro in null allele *Bmal1−/−* models (Edgar et al., 2016). MuHV-4 DNA levels were higher when inoculation occurred at the beginning of the resting phase compared with infecting at the start of the active phase. Studies with a bioluminescent reporter virus showed an increased frequency of infected cells when *Bmal1* expression was high. Of note, MuHV-4 induced *Bmal1* expression regardless of the time of infection, suggesting that herpesviruses can perturb cellular circadian cycling. Similar observations were reported in HSV-1 and influenza A infection (Edgar et al., 2016).

Two independent studies assessed the circadian regulation of influenza virus infection (Ehlers et al., 2018; Sengupta et al., 2019). Both groups reported that *Bmal1* KO mice showed greater asthma-like airway changes and worse acute viral bronchitis (Ehlers et al., 2018), and survival was higher when mice were infected before their active phase compared with before the resting phase (Sengupta et al., 2019). In contrast to the earlier Edgar study, Sengupta et al. did not observe differences in viral load when infecting mice at different time points. Infection at the start of the active phase promoted lung inflammation independent of the viral burden, suggesting that the more severe outcome of influenza infection is mediated by time-dependent regulation of host tolerance and immune activation pathways. Together, these studies highlight the importance of model systems in the viral-circadian studies—the in vitro isolated culture or the in vivo multicellular interactions—to characterize the role of circadian components in the viral life cycle.

## Viruses and the Circadian CLOCK Shape the Chromatin Landscape

**Figure 3.**
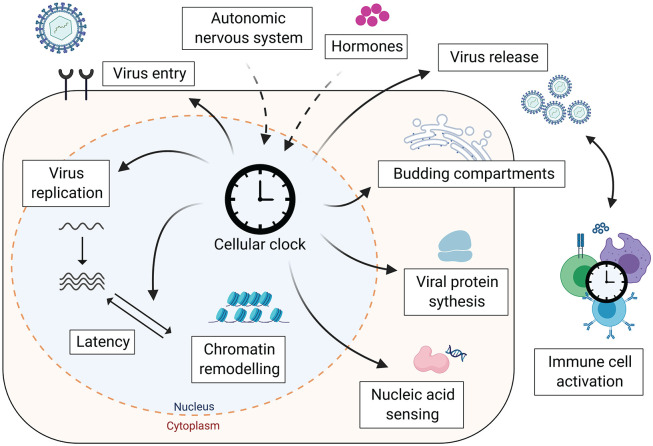
Cellular clock affects the virus life cycle. The cellular clock can influence viral infection at multiple steps in the virus life cycle, from the regulation of entry receptors to lipid-dependent pathways for particle genesis. In addition, immune responses combating viral infections, such as nucleic acid sensing and the activity of immune cells, are regulated by cellular circadian clocks. Created with BioRender.com

The influence of the circadian clock machinery on epigenetic regulation and chromatin remodeling partly reflects the histone deacetylase activity of CLOCK (Doi et al., 2006; Aguilar-Arnal and Sassone-Corsi, 2015). Time-dependent binding of circadian transcription factors (TFs) (Chen et al., 2015; Y. Xu et al., 2016) influences the chromatin state and RNA Pol II recruitment (Koike et al., 2012; Le Martelot et al., 2012; Pacheco-Bernal et al., 2019). Genome-wide RNA polymerase II profiles revealed rhythmic Pol II recruitment at promoters and dynamic changes in histone marks suggest daily remodeling of the epigenetic landscape (Le Martelot et al., 2012). A single circadian factor can control opposing transcriptional phases to generate complex circadian rhythms with multiple phases of gene expression (Fang et al., 2014). Clock components can define the transcriptionally permissive chromatin landscape that regulates the accessibility and activity of host transcriptional machinery (Menet et al., 2014; Trott and Menet, 2018). Viruses with DNA genomes may exploit this circadian epigenetic machinery to promote their own transcription and replication (Figure 3). HSV establishes a latent infection with periodical reactivation (Nicoll et al., 2012) and induces *Bmal1* expression, leading to the reprogramming of clock signaling pathways. Kalamvoki and Roizman showed that the HSV-encoded protein ICP0 interacts with the CLOCK:BMAL1 histone acetyl transferase complex, and infecting cells with small interfering RNA (siRNA)-silenced *Clock* expression or engineered to express *Clock* mutants significantly reduced viral replication (Kalamvoki and Roizman, 2010; Kalamvoki and Roizman, 2011). These findings support a model where herpesvirus interaction with circadian clock components leads to a remodeling of viral chromatin that can regulate latency.

## Circadian and Annual Rhythms Affecting Viral Transmission

The mammalian circadian rhythm adapts to seasonal changes in the natural light/dark cycle; thus, the link between viral infections and circadian clock goes beyond the 24-h period. Seasonal differences may explain the increased susceptibility to viral infections during the winter months (Dowell, 2001). Annual cyclic incidence is a feature of epidemic disease, which is driven by environmental and exogenous factors, as well as host behavior and phenology (Martinez, 2018). Gene expression by immune cells and the cellular composition of blood varies by season, with gene expression patterns inverted between Northern and Southern hemispheres. Disease risk biomarkers like IL-6 receptor and C-reactive protein show increased expression in winter, which coincides with lower BMAL1 expression (Dopico et al., 2015). Similarly, immune function shows seasonal variation, with decreased neutrophil activity (Klink et al., 2012) but increased total blood monocyte numbers during winter (Dopico et al., 2015). An important regulator of seasonal physiological changes is altered hormone levels, where thyroid hormone levels vary in the hypothalamus and glucocorticoid receptors show their lowest expression in winter (Wood and Loudon, 2014; Dopico et al., 2015). The host, as well as nonhuman virus reservoirs and vector rhythmicity, impacts acute and chronic diseases outcomes.

Many viruses are transmitted by insect vectors that have their own circadian rhythm and are active at different times of the day (Meireles-Filho and Kyriacou, 2013). This influences whether the virus will encounter the host in its active or resting phase. For example, dengue virus is transmitted to humans by *Aedes aegypti* mosquitoes that are active during the day and dusk/dawn, and virus infection enhances mosquito activity (Lima-Camara et al., 2011). The transmission of Zika virus (ZIKV) is thought to be dependent on seasonal climate changes, and as more ZIKV incidence data become available, it will be important to define high and low transmission seasons (Petersen et al., 2016). Seasonally adjusted planning of pregnancy could limit vertical transmission by ensuring that sensitive gestational periods do not coincide with the ZIKV season (Martinez, 2016).

Human agricultural and technological interventions may also enhance the spread of viral diseases, for example, rice irrigation in summer months was shown to increase the incidence of Japanese encephalitis virus infection (Tian et al., 2015). Winter-associated light pollution is associated with increased risk of West Nile virus infection (Kernbach et al., 2019). Finally, climate change is altering annual environmental rhythms and disrupting seasonal biology, which may desynchronize biological clocks and impact human health (Stevenson et al., 2015).

## Viruses Dictating the Time

Just as the host circadian rhythm can influence virus replication, virus infection can perturb the host circadian system (Figure 3). In vitro studies show that the HBV-encoded regulatory protein HBx perturbed circadian-regulated gene transcripts (Yang et al., 2014). It is worth noting that this study used HBx overexpression systems, and so these conclusions will require validation in authentic viral replication models. Levels of the microRNA miR-10a were elevated in liver biopsies from chronic HCV patients (Horii et al., 2019). In vitro analysis showed that overexpressing miR-10a in hepatocytes reduced *Bmal1* expression by suppressing RORα. In a separate study, in vitro cell models expressing HCV core protein and liver biopsies from HCV-infected patients showed reduced PER2 and CRY2 expression (Benegiamo et al., 2013). Viral-dependent disruption of circadian signaling pathways may contribute to liver disease (Mukherji et al., 2019).

Influenza virus altered the timing of *Bmal1, Clock*, and *Rev-erbβ* peak expression in the lungs of infected mice and reduced the amplitude of *Per2* expression in lung tissue explants (Sundar et al., 2015). Combined smoke exposure and influenza infection reduced locomotor activity in mice and has been associated with more severe asthma in humans (Ehlers et al., 2018). Simian immunodeficiency virus was reported to impair the amplitude and mean of the circadian rhythm of body temperature in monkeys and reduce locomotor activity (Huitron-Resendiz et al., 2007). HIV has also been shown to alter the circadian system in mice and humans. The HIV-encoded transactivator of transcription (Tat) protein resets the murine circadian clock by influencing light entrainment pathways (Clark et al., 2005). Chronic overexpression of Tat decreased the amplitude of the circadian wheel-running rhythm and locomotor activity in mice (Duncan et al., 2008). In HIV-1-infected patients, the Tat protein has been reported to promote melatonin expression, which may contribute to the impaired sleep quality frequently reported by HIV-infected individuals (Wang et al., 2014). In summary, viruses can influence circadian gene expression level by altering the activity of clock transcription factors or more generally via perturbing hormonal and light entrainment pathways. Published studies have largely focused on the direct effect(s) of viral-encoded proteins to interact with circadian regulators; however, additional pathways are likely to play a role and require investigation. For example, viruses that integrate into the host chromatin as part of their life cycle may perturb circadian-regulated gene expression. Many open questions remain, which would be interesting to investigate with a circadian lens in future studies.

## Perfect Timing: Approaches for Therapy

### Circadian Modulators

The host circadian machinery provides a wealth of therapeutic targets for antiviral interventions. Synthetic agonists of REV-ERB show in vivo activity in murine models (Solt et al., 2012) and alter immune responses by selective regulation of inflammatory cytokines (Gibbs et al., 2012). These agonists were reported to inhibit HCV entry, RNA replication, and release of infectious particles (Zhuang et al., 2019) via repressing expression of the fatty acid biosynthesis pathway component stearoyl-CoA-desaturase (SCD), a rate-limiting enzyme for HCV replication (Lyn et al., 2014; Nio et al., 2016). Similar observations were seen with the related flaviviruses, DENV and ZIKV, that also depend on fatty acid biosynthesis pathways (Paul and Bartenschlager, 2015). More recently, REV-ERB agonists were shown to inhibit HIV transcription and virus replication in primary cells (Borrmann et al., 2020). HSV and HIV can establish latent infection and thereby evade antiviral drugs and host immune responses. The histone deacetylase activity of the clock components could be targeted by circadian-modifying drugs to activate latent virus, complementing existing “shock and kill” eradication approaches for curing HIV (Deeks, 2012).

Additional pharmacological agents that modulate circadian protein activity have been developed (Miller and Hirota, 2020). These include CRY stabilizers (Hirota et al., 2012; J. W. Lee et al., 2015a) that inhibit BMAL1 expression and ROR modulators (Huh et al., 2011; Solt et al., 2011; Chai et al., 2020). ROR inverse agonists (Kumar et al., 2011) could have similar effects on viral replication as REV-ERB agonists, since they compete and bind the same DNA response element and regulate many of the same genes (Takeda et al., 2012). These ligands could be beneficial under certain circumstances to enhance or inhibit inflammatory (T. Xu et al., 2011) and viral responses.

It is interesting to consider the impact of circadian gene polymorphism on virus infection. K. A. Lee et al. (2015b) observed an association between genetic variability in circadian genes and sleep patterns in adults with HIV. In HIV-infected individuals, polymorphism in *Clock* and *Per2* genes was associated with poor sleep maintenance (wake after sleep onset) and increased total sleep time; however, the underlying mechanisms are not well defined (K. A. Lee et al., 2015b).

### Time of Day, Drug Administration, and Vaccination

Optimizing the time of day for medication is one aspect of personalized medicine, illustrated by the circadian timing of anticancer treatments (X. M. Li et al., 2013). An improved understanding of the circadian system may alter the time and amount of anti- viral drugs required. Recent studies show that the effective dose of acyclovir required to prevent HSV-2 infection in mice is 4 times higher during the active phase than during rest (Matsuzawa et al., 2018). Drug half-life is dependent on dosing time, such that drugs with short half-lives (6 h or less) may be more sensitive to the time of day of administration (Ruben et al., 2019).

The circadian clock of CD8 T cells modulates their response to vaccination, leading to higher T-cell activation in mice when vaccination with dendritic cells preloaded with ovalbumin peptide was performed during the day compared with during the night (Nobis et al., 2019). Influenza vaccination in humans was more effective when administered in the morning (9-11 a.m.) compared with in the afternoon (3-5 p.m.), with higher antibody responses measured against multiple influenza strains. No associations were observed between sex, steroid hormone or cytokine levels, and antibody responses (Long et al., 2016). The month of vaccine administration in countries with seasonally dependent environments can also affect antibody responses and protection against a range of viruses. The expression of B-cell maturation factor (BCMA or TNFRSF17) shows seasonal variation (Dopico et al., 2015) and expression levels are associated with a favorable response to the trivalent influenza vaccine (Nakaya et al., 2011; S. Li et al., 2014). Significant associations were observed between month of vaccination and antibody response to rabies vaccines in Pakistan compared with in Gambia (Moore et al., 2006). Seasonality in PRR expression that show increased expression during the winter months (Dopico et al., 2015) alters the quality of vaccine responses and correlates with better protection against yellow fever virus (Querec et al., 2009). These simple measures of timing drug or vaccine administration can increase drug efficiency and reduce side effects (Ruben et al., 2019; Zhao et al., 2020).

## Conclusions and Keeping Track of Time

Having reviewed this growing field, we find clear evidence for a link between circadian rhythms and viral infection. Studying the interplay between circadian pathways and virus replication can offer many opportunities: a better understanding of viral infections and related immune responses, identification of new antiviral targets, refinement of current therapies, and treatment of chronic infection. The circadian clock is connected to many other pathways, including hypoxia signaling (Adamovich et al., 2017; Manella et al., 2020; Peek, 2020) and metabolism (Panda, 2016; Alexander et al., 2020; Cal-Kayitmazbatir et al., 2020). This crosstalk is likely to influence viral infection, and understanding these interactions will require multidisciplinary approaches involving virology, circadian biology, immunology, and pharmacology. Evolution is defined by the convergence of external rhythms with internal biology. As we live in an era of shift work–related sleep disorders, social jet lag, and global viral pandemics, together with emergent viral drug resistance, exploiting biological rhythms could provide novel treatment modalities and new drugs for treating viral infections. Plato wrote that “Rhythm and harmony find their way into the inward places of the soul” (Plato, The Republic, c. 375 BC): it is our responsibility to study how rhythmicity and synchronization can be integrated into human health.
